# Choice Bundling Increases Valuation of Delayed Losses More Than Gains in Cigarette Smokers

**DOI:** 10.3389/fnbeh.2021.796502

**Published:** 2022-01-13

**Authors:** Jeffrey S. Stein, Jeremiah M. Brown, Allison N. Tegge, Roberta Freitas-Lemos, Mikhail N. Koffarnus, Warren K. Bickel, Gregory J. Madden

**Affiliations:** ^1^Fralin Biomedical Research Institute at VTC, Roanoke, VA, United States; ^2^Department of Human Nutrition, Foods, and Exercise, Virginia Tech, Blacksburg, VA, United States; ^3^Department of Family and Community Medicine, University of Kentucky, Lexington, KY, United States; ^4^Department of Psychology, Utah State University, Logan, UT, United States

**Keywords:** choice bundling, sign effect, intertemporal choice, delay discounting, cigarette smoking, impulsive choice, choice bracketing

## Abstract

Choice bundling, in which a single choice produces a series of repeating consequences over time, increases valuation of delayed monetary and non-monetary gains. Interventions derived from this manipulation may be an effective method for mitigating the elevated delay discounting rates observed in cigarette smokers. No prior work, however, has investigated whether the effects of choice bundling generalize to reward losses. In the present study, an online panel of cigarette smokers (*N* = 302), recruited using survey firms Ipsos and InnovateMR, completed assessments for either monetary gains or losses (randomly assigned). In Step 1, participants completed a delay-discounting task to establish Effective Delay 50 (ED50), or the delay required for an outcome to lose half of its value. In Step 2, participants completed three conditions of an adjusting-amount task, choosing between a smaller, sooner (SS) adjusting amount and a larger, later (LL) fixed amount. The bundle size (i.e., number of consequences) was manipulated across conditions, where a single choice produced either 1 (control), 3, or 9 consequences over time (ascending/descending order counterbalanced). The delay to the first LL amount in each condition, as well as the intervals between all additional SS and LL amounts (where applicable), were set to individual participants’ ED50 values from Step 1 to control for differences in discounting of gains and losses. Results from Step 1 showed significantly higher ED50 values (i.e., less discounting) for losses compared to gains (*p* < 0.001). Results from Step 2 showed that choice bundling significantly increased valuation of both LL gains and losses (*p* < 0.001), although effects were significantly greater for losses (*p* < 0.01). Sensitivity analyses replicated these conclusions. Future research should examine the potential clinical utility of choice bundling, such as development of motivational interventions that emphasize both the bundled health gains associated with smoking cessation and the health losses associated with continued smoking.

## Introduction

Behavioral outcomes are devalued as a function of the delay until they are experienced (for review, see Odum, 2011). This process, known as delay discounting, is reliably associated with cigarette smoking (for meta-analysis, see MacKillop et al., 2011; Amlung et al., 2016) and other tobacco use (e.g., Stein et al., 2018a; DeHart et al., 2020). For example, high discounting rates for delayed monetary gains are cross-sectionally associated with smoking status (e.g., Mitchell, 1999) and longitudinally predict both smoking initiation (Audrain-McGovern et al., 2009) and relapse following smoking cessation treatment (e.g., Yoon et al., 2007; Sheffer et al., 2014). These findings indicate that delay discounting is a potential therapeutic target in tobacco cessation (Riddle and Science of Behavior Change Working Group, 2015), in which interventions that increase valuation of delayed outcomes may also reduce cigarette smoking (e.g., Stein et al., 2016, 2018b; Chiou and Wu, 2017). Thus, understanding how delay discounting influences intertemporal choice between smaller, sooner (SS) and larger, later (LL) outcomes is critical and may lead to efficacious interventions for tobacco use.

A large and growing literature has explored the effects of various behavioral, pharmacological, and neurocognitive interventions on delay discounting. The intertemporal choices arranged in these studies involve economic gains, such as monetary and food rewards (for reviews, see Perry and Carroll, 2008; Bickel et al., 2017; Rung and Madden, 2018). However, remarkably few studies have explored the effects of these interventions on choices involving economic losses. This is concerning, as at least three sets of findings suggest that intervention effects on gains may not generalize to losses. First, losses are discounted at a lower rate than gains of an equivalent size (i.e., the “sign effect”; Thaler, 1981; Benzion et al., 1989; Murphy et al., 2001; Estle et al., 2006); thus, interventions may prove ineffective with losses due to a ceiling effect. Second, although discounting of losses is associated with cigarette smoking in a manner similar to that of gains (Odum et al., 2002; Baker et al., 2003; Johnson et al., 2007), prior research reveals mixed findings on whether discounting rates for gains and losses are correlated (Chapman, 1996; Hardisty and Weber, 2009; Mitchell and Wilson, 2010; Harris, 2012). Third, and finally, the commonly reported inverse relationship between discount rate and amount of the outcome (i.e., the “magnitude effect”) appears less robust for losses than for gains (Estle et al., 2006; Mitchell and Wilson, 2010; Green et al., 2014). Collectively, these findings suggest the presence of one or more processes, secondary to discounting, that differ between valuation of delayed gains and losses. Thus, further research investigating whether valuation of delayed gains and losses are amenable to the same interventions is warranted. Knowledge gained from these studies may help guide whether clinical interventions for tobacco use should focus on enhancing sensitivity to the delayed health gains associated with smoking cessation, the delayed health losses associated with continued smoking, or both.

### Delay Discounting of Gains and Losses

The extent to which the value of an outcome (gain or loss) is devalued with increasing delay is generally well-described by the following hyperbolic form (Mazur, 1987):


(1) $$V=\frac{A}{1+kD}$$

where *V* is the subjective value of an outcome, *A* is its objective amount, *D* is its delay, and *k* is a free parameter that describes the nonlinear rate of discounting. This model may be used to predict intertemporal choice between SS and LL outcomes. When the outcomes are gains, choice is allocated to the option that maximizes subjective benefit. In contrast, when the outcomes are losses, choice is allocated to the option that minimizes subjective harm.

Consider a choice between receiving either $450 now or $900 in 1 year. The SS gain is available immediately and, thus, is not discounted—its subjective value is equal to its nominal value ($450). In contrast, the LL gain ($900) is discounted according to the prevailing value of *k* (determined by both trait and state factors; Odum and Baumann, 2010). If *k* = 0.003, for example, the subjective value (*V*) from Equation 1 of the $900 LL reward would be $429.59. Here, preference for the SS monetary gain is predicted because it provides a larger gain ($450) than the subjective value of the LL option ($429.59). In contrast, if this same choice were instead between *losing* either $450 now or $900 in 1 year, then preference for the LL option is predicted because it minimizes subjective loss ($429.59) compared to the SS option ($450).

### Choice Bundling of Gains and Losses

As originally noted by Ainslie (1975, 2001), a prediction unique to hyperbolic (as opposed to exponential) delay discounting is that conditions in which a single choice produces a series of repeating SS or LL outcomes (i.e., a choice bundle) can increase relative preference for the LL option compared to equivalent choices for unbundled (single) outcomes. This is due to the non-constant rate of devaluation in hyperbolic discounting in which value is lost quickly at short delays and more slowly at long delays. When a single choice produces repeating outcomes, the relatively stable subjective values of individual LL outcomes sum to a larger value than the sum of individual SS outcomes (for further discussion, see Ainslie, 2001; Ashe and Wilson, 2020; Stein and Madden, 2021).

This effect of choice bundling on delay discounting is predicted quantitatively by an additive model of hyperbolic discounting (Mazur, 1986, 1989):


(2) $$V_{bundle}=\sum_{i=1}^{n}\left(\frac{A}{1+kD}\right)$$

in which the subjective value of a bundled series of outcomes (*V*_*bundle*_) is equal to the summed values of all rewards in the bundle (all parameters are as described for Equation 1). For example, consider again the (unbundled) choice between receiving $450 now or $900 in 1 year. When *k* = 0.003, choice for the SS option is predicted because its undiscounted value ($450) exceeds the subjective value of the LL option ($429.59). In contrast, if these same amounts ($450 and $900) were distributed equally across a series of repeating rewards and the choice were instead between receiving either (a) a SS bundle of $150 now, $150 in 1 year, and $150 in 2 years or (b) a LL bundle of $300 in 1 year, $300 in 2 years, and $300 in 3 years, the summed subjective values (*V*_*bundle*_) of the SS and LL options would now be $268.62 and $307.25, respectively. Thus, choice bundling is predicted to shift preference toward the LL over the SS gains, even though neither the absolute nor the relative differences between these amounts have changed. Likewise, if this same choice were instead between bundled losses, choice bundling is predicted to shift preference toward the SS over the LL losses.

Several studies have offered empirical support for this predicted effect of choice bundling (for review, see Ashe and Wilson, 2020), showing that bundling increases preference for LL gains (money and/or food) in both humans (Kirby and Guastello, 2001; Hofmeyr et al., 2011) and nonhumans (Ainslie and Monterosso, 2003; Stein et al., 2013). These include a recent study from our group (Stein and Madden, 2021) showing in an online sample from the general population (*N* = 252) that the efficacy of choice bundling is enhanced by increasing the number of rewards in the choice bundle (for a similar finding in rats, see Stein et al., 2013). Observed effects in this online study also approximated those predicted by the additive model of hyperbolic discounting (Equation 2).

Despite these encouraging results, more work remains to be done. Specifically, no prior studies have investigated if comparable effects of choice bundling can be achieved with losses. Equation 2 makes no distinction between gains and losses but, as previously noted, some degree of discordance characterizes the discounting of delayed gains and losses (i.e., the “sign effect”; Thaler, 1981; Benzion et al., 1989; Murphy et al., 2001; Estle et al., 2006). In addition, only one study to our knowledge has investigated choice bundling in clinical populations (cigarette smokers; Hofmeyr et al., 2011) who may benefit from interventions to mitigate high discounting rates. Accordingly, the present study used a mixed between- and within-subjects design to examine the effects of choice bundling on valuation of delayed gains and losses in an online panel of cigarette smokers. We did so using an adaptive, two-step procedure in which we: (1) assessed delay discounting to establish individual participants’ values of Effective Delay 50 (ED50), or the delay required for an outcome to lose half of its value (Yoon and Higgins, 2008), and (2) used these participant-specific ED50 values to inform the delays experienced in the choice bundling assessments in order to control for differences in discounting of gains and losses.

## Materials and Methods

A US-based panel of cigarette smokers (*N* = 308) were recruited by market research firms, Ipsos (iSay panel^1^) and InnovateMR,^2^ for a separate parent study examining the effects of cigarette and nicotine vaping product flavor restrictions on hypothetical tobacco product purchasing. Ipsos distributed the survey to panelists in July and August 2021. Both the parent and present study were registered on www.clinicaltrials.gov (NCT05110872 and NCT05110716, respectively). Participants first completed a brief screening questionnaire in which they reported their smoking history, current smoking status, usual brand of cigarette, and age. To be eligible for both the parent and present study, participants were required to: (1) currently smoke at least 10 cigarettes per day, (2) have smoked at least 100 cigarettes in their lifetime, and (3) be 21 years of age or older. Menthol and non-menthol smokers were recruited in approximately equal numbers.

After screening, eligible participants first completed procedures for the parent study. These included multiple conditions in the Experimental Tobacco Marketplace (for review, see Bickel et al., 2018), in which they made hypothetical tobacco product purchases while the price of cigarettes and the available tobacco product flavors were varied. Following completion of these procedures, participants were randomly assigned in the present study to complete assessments for either monetary gains (*n* = 155) or losses (*n* = 153) and completed relevant procedures, described below (section “Procedures”).

**FIGURE 1:**
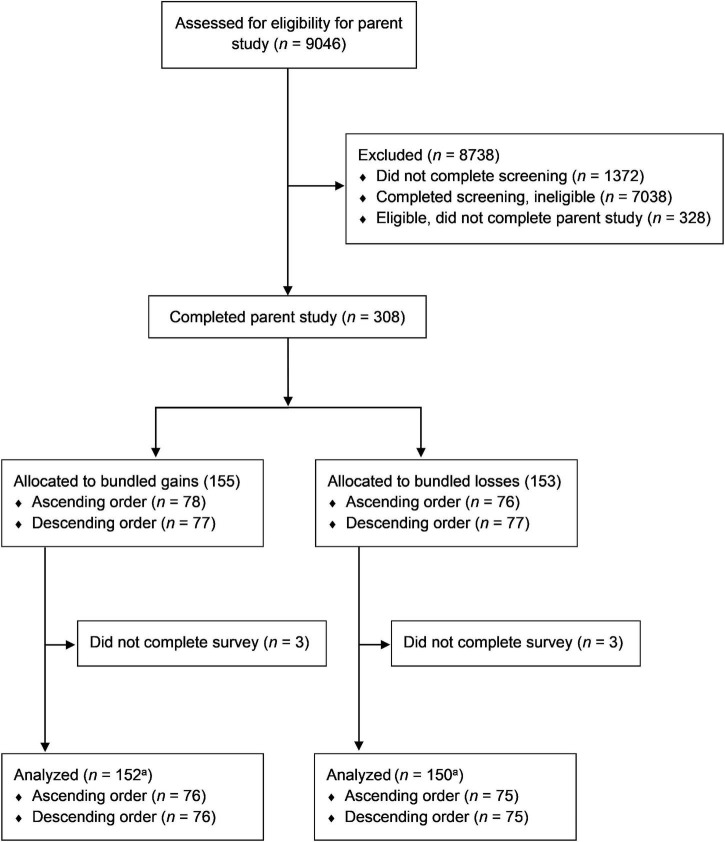
CONSORT flow diagram showing participant flow through study screening, group allocation, and data analysis. ^a^Denotes sample size in primary analysis. Subsets of this sample were used in sensitivity analyses (see text for details).

The numbers of participants who screened for, completed, and were analyzed for the present study are provided in Figure 1. Note that six participants did not complete the study (*n* = 3 each from gains and losses groups), leaving a final analytic sample of *N* = 302. Participants required a median time of 36.95 min to complete the full survey (interquartile range: 29.47–55.78) and received the equivalent of $8.57–$14.28 of monetary compensation in the form of virtual currencies (e.g., online and mobile gift cards).

### Procedures

Study procedures were implemented using Qualtrics online survey software (Qualtrics, Provo, UT, United States). All procedures were reviewed and approved by the Virginia Tech Institutional Review Board. Informed consent was implied through completion of the survey.

#### Step 1: Assessment of Delay Discounting

Delay discounting was assessed using a version of the recently developed six-trial, adjusting-delay task (Koffarnus et al., 2021). This task was modified from the similar and commonly used five-trial, adjusting-delay task (Koffarnus and Bickel, 2014) to provide greater range and resolution in measurement of ED50 (and *k*). Specifically, whereas the original five-trial task provides the ability to measure only 32 possible ED50 values ranging from 1 h to 25 years, the six-trial task allows measurement of 64 possible ED50 values. In the version used in the present study, possible ED50 values ranged from 4 s to 90 years in approximately logarithmic intervals. We note, however, that these values in the original version of the six-trial task developed by Koffarnus et al. (2021) range from 5 s to 65 years.

Participants were presented with repeated, hypothetical choices between receiving or losing (depending on group) a larger amount ($900) after a delay and half of this amount ($450) immediately. The delay to the LL amount started at 1 day on the first trial and was adjusted following each trial, based on the preceding choice. Specifically, in the gains task, choices for the larger amount increased the delay, and choices for the smaller amount decreased the delay, on the next trial; in the losses task, this relationship was reversed. The adjusted value after the final trial was the delay expected to produce indifference between options and provided a measure of ED50. Higher values of ED50 reflect less discounting of the delayed outcome. Additional details regarding this task, including the task instructions, logic for trial branching, and method for scoring ED50 and *k*, are provided in Supplementary Material.

#### Step 2: Assessment of Choice Bundling Effects

Participants completed three conditions of an adjusting-amount task, modified from those used previously (Du et al., 2002; Athamneh et al., 2017) to allow assessment of choice bundling effects. The instructions participants read prior to the task are provided in Supplementary Material.

**FIGURE 2:**
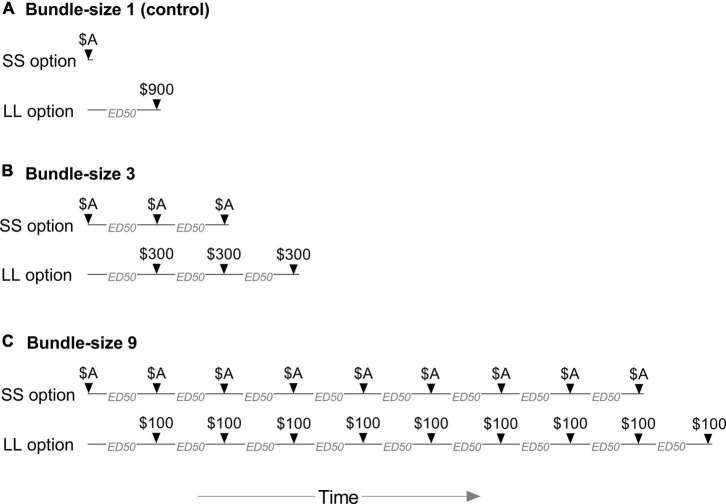
Choices in the adjusting-amount task between the smaller, sooner (SS) and larger, later (LL) options in the bundle-size 1 (control), 3, and 9 conditions. The total monetary value (gain or loss, depending on group) of the SS adjusting amount (A) started at $450 on the first trial and was adjusted after each trial until reaching an indifference point. The delays to the first LL consequence, as well as the intervals between all SS and LL consequences, were set to individual participants’ Effective Delay 50 (ED50) values from the six-trial, adjusting-delay task.

As depicted in Figure 2, the bundle size (i.e., number of consequences) was manipulated across conditions, where a single choice produced either 1 (control), 3, or 9 outcomes (gains or losses) over time. Participants completed these bundle-size conditions in either ascending or descending order (counterbalanced). Each condition featured six trials in which participants chose between receiving or losing (depending on group) either LL fixed amounts or adjusting SS amounts. The total amount of the LL option equaled $900. This value was divided equally among all gains/losses in the bundle (see Figure 2) in order to hold the amounts constant across bundle-size conditions. This procedure controlled for the “magnitude effect” in delay discounting research, in which degree of discounting is inversely related to amount (e.g., Estle et al., 2006; for further discussion relevant to choice bundling, see Stein and Madden, 2021).

The total SS amount was also divided equally among all gains/losses in the bundle. This value started at $450 on the first trial in each condition and was adjusted following each of six trials, based on the preceding choice, according to procedures described previously (Du et al., 2002). In the gains task, choices for the LL option increased the total SS amount, and choices for the SS option decreased the total SS amount, on the next trial. In the losses task, this relationship was again reversed. The size of these adjustments (up or down) started at $225 after the first trial (half of the SS amount) and was reduced by half at each subsequent trial ($112.50 after the second trial, $56.25 after the third trial, etc.). The total adjusted amount after the final, sixth trial served as the indifference point, with higher values reflecting greater valuation of the LL option.

The delay to the first LL amount in each condition, as well as the intervals between all additional SS and LL amounts (where applicable), were set to individual participants’ ED50 values from Step 1. This maximized the probability that indifference points in the bundle-size 1 control condition would be near the $450 midpoint. Moreover, in the bundle-size 3 and 9 conditions, this ensured that the intervals between all contiguous gains or losses were directly proportional to participants’ baseline level of delay discounting. This was done to both control for differences in discounting of gains and losses (e.g., Green et al., 1997) and to provide approximately equal sensitivity to detect both increases and decreases in valuation of the LL option.

#### Data Quality

Neither the six-trial, adjusting-delay task nor the use of the adjusting-amount task in this study allowed application of standardized criteria to detect nonsystematic responding (Johnson and Bickel, 2008). To mitigate this concern, data quality in the choice bundling assessment was monitored by inclusion of three quality control questions, similar to methods used previously (Stein et al., 2018a; Stein and Madden, 2021). Specifically, after the sixth trial in each of three adjusting-amount conditions, a seventh trial asked participants to choose between $450 now and $900 now. In the bundle-size 3 and 9 conditions, these monetary amounts were framed as separate rewards, as described above for these conditions; however, all delays were removed (e.g., $300 now, $300 now, and $300 now; Stein and Madden, 2021). Choice of the smaller option in these questions was interpreted as inattention or atypical valuation of monetary rewards.

#### Demographic and Smoking Characteristics

At the end of the survey, participants completed a demographics questionnaire and the Heaviness of Smoking Index (Heatherton et al., 1989).

### Data Analysis

All analyses were performed in SPSS version 27.0 (IBM SPSS Statistics for Windows, IBM Corp.). The final analytic sample (*N* = 302) provided 95% power to detect an approximately small effect size (*f* = 0.108) within-subjects x between-groups interaction in analysis of variance (ANOVA), assuming three repeated measures, four groups, a = 0.05, and a correlation between repeated measures of *r* = 0.50.

#### Participant Characteristics

Demographic, smoking, and other sample characteristics were compared between the four groups (gains/ascending, gains/descending, losses/ascending, and losses/descending) using two-way ANOVA, Fisher’s Exact tests, or logistic regression.

#### Step 1: Assessment of Delay Discounting in the Six-Trial, Adjusting-Delay Task

Effective Delay 50 values were compared between the gains and losses group using one-way analysis of covariance (ANCOVA), with sign (gain, loss) as a between-subjects factor. ED50 values were nonnormally distributed and were thus log (base 10) transformed prior to analysis.

#### Step 2: Effects of Choice Bundling in the Adjusting-Amount Task

Indifference points in the bundle-size 1, 3, and 9 conditions were analyzed using repeated-measures ANCOVA, with bundle size as a within-subjects factor and sign (gain, loss) and order (ascending, descending) as between-subjects factors. Significant results were followed by between-group and within-subject post-hoc comparisons. Bonferroni correction was used to maintain the family-wise error rate in each post-hoc test at *a* = 0.05.

#### Sensitivity Analyses

Analysis of covariances described above were repeated in sensitivity analyses when excluding: (1) participants who failed one or more of the quality control questions and (2) participants whose ED50 values in Step 1 produced unrealistic delays to one or more bundled consequences in Step 2 (described further, below).

## Results

### Participant Characteristics

Table 1 provides demographic characteristics for gains and losses groups, by bundle-size order (ascending and descending). On average, participants smoked 19.43 cigarettes/day (±11.78) and were 48.89 years old (±13.66). The sample was exactly 50% male and female. The majority were white (89.7%), non-Hispanic (84.8%), and reported low (i.e., <$50,000/year; 49.3%) or middle (i.e., $50,000–$150,000/year; 40.7%) incomes.

**TABLE 1:** Demographic and smoking characteristics.

Sign	Gains		Losses	
Bundle-size order	Ascending	Descending	Ascending	Descending
*n*	76	76	75	75
**Demographics**				
Age (year; ± SD)	49.6 ± 14.5	47.2 ± 12.6	49.7 ± 13.9	49.1 ± 13.6
Gender				
% Male (*n*)	53.9 (41)	42.1 (32)	52.0 (39)	52.0 (39)
% Female (*n*)	46.1 (35)	57.9 (44)	48.0 (36)	48.0 (36)
Race				
% White (*n*)	89.5 (68)	86.8 (66)	94.7 (71)	89.2 (66)
% Asian (*n*)	3.9 (3)	0.0 (0)	0.0 (0)	1.3 (1)
% Black/African American (*n*)	5.3 (4)	10.5 (8)	5.3 (4)	1.3 (1)
% Other race or multi-racial (*n*)	1.3 (1)	2.6 (2)	0.0 (0)	8.1 (6)
% Not answered (*n*)	0.0 (0	0.0 (0	0.0 (0	1.3 (1)
Ethnicity				
% Non-Hispanic/Latino (*n*)	73.7 (56)	85.5 (65)	90.7 (68)	89.3 (67)
% Hispanic/Latino (*n*)	25.0 (19)	13.2 (10)	8.0 (6)	9.3 (7)
% Not answered (*n*)	1.3 (1)	1.3 (1)	1.3 (1)	1.3 (1)
Household income				46.7 (35)
% <$50k (*n*)	46.1 (35)	56.6 (43)	48.0 (36)	41.3 (31)
% $50k-$149,999 (*n*)	39.5 (30)	34.2 (26)	48.0 (36)	12.0 (9)
% ≥$150k (*n*)	14.5 (11)	9.2 (7)	4.0 (3)	
Education				
% ≤High school (*n*)	30.3 (23)	35.5 (27)	22.7 (17)	30.7 (23)
% Some college (*n*)	28.9 (22)	40.8 (31)	36.0 (27)	29.3 (22)
% ≥4-year college degree (*n*)	40.8 (31)	23.7 (18)	40.0 (30)	40.0 (30)
**Smoking characteristics**				
Cigarettes/day ( ± SD)	20.9 ± 14.6	19.5 ± 8.8	17.7 ± 7.9	19.8 ± 14.5
HSI ( ± SD)	3.2 ± 1.5	3.4 ± 1.3	3.0 ± 1.4	3.2 ± 1.2
Usual brand flavor				
% Menthol (*n*)	47.4 (36)	43.4 (33)	48.0 (36)	46.7 (35)

*Cigarettes/day reflects daily cigarette consumption in the month preceding the survey.*

No participant characteristics differed significantly between groups (*ps* > 0.05), with the exception of ethnicity. Participants reporting Hispanic/Latino ethnicity were sampled less frequently in the losses group (10.0%) compared to the gains group (20.39%), OR = 0.261 (0.098, 0.697), *p* = 0.007, though likely due to chance because the groups were randomized. No significant associations were observed between ethnicity and either order or the Sign × Order interaction (in both cases, *ps* > 0.05). Ethnicity was included as a covariate in ANCOVA, described below.

### Step 1: Assessment of Delay Discounting in the Six-Trial, Adjusting-Delay Task

**FIGURE 3:**
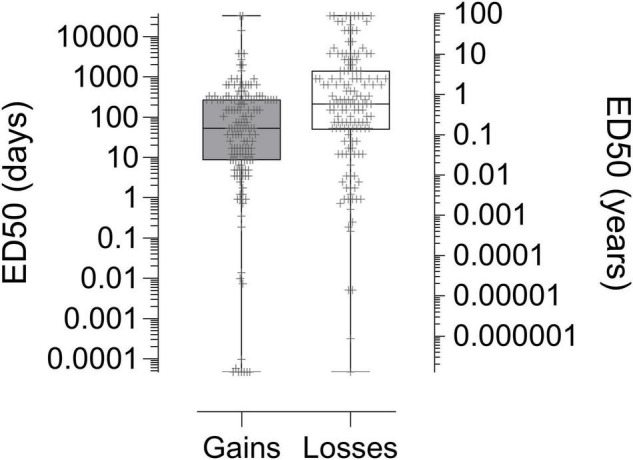
Median Effective Delay 50 (ED50) values in the six-trial, adjusting-delay task for monetary gains (*n* = 152) and losses (*n* = 150), scaled in both days (left *y* axis) and corresponding years (right *y* axis). Higher values of ED50 reflect less discounting of the delayed gain or loss. Significantly higher ED50s were observed for losses compared to gains (*p* < 0.001).

Analysis of log transformed values revealed higher ED50s (i.e., lower discounting) for delayed losses compared to gains, *F*(1, 299) = 14.008, *p* < 0.001; η_p_^2^ = 0.045 (see Figure 3). Ethnicity was a significant covariate, *F*(1, 299) = 3.940, *p* = 0.048; η_p_^2^ = 0.013, with lower ED50 values (i.e., greater discounting) observed in participants reporting Hispanic/Latino ethnicity.

### Step 2: Assessment of Choice Bundling Effects in the Adjusting-Amount Task

**FIGURE 4:**
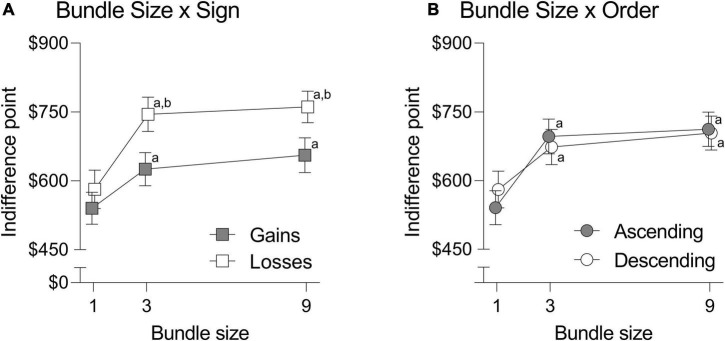
Significant effects of the Bundle Size x Sign **(A)** and Bundle Size x Order **(B)** interactions on indifference points in the adjusting-amount task. Error bars reflect 95% confidence intervals. Higher indifference points reflect greater valuation of the LL option. ^a^Significantly different from bundle-size 1 within the same sign or order group, *p* < 0.001. ^b^Significantly different from the opposing sign or order group at the same bundle size, *p* < 0.001.

Analysis of covariance revealed significant main effects of bundle size, *F*(2, 594) = 10.793, *p* < 0.001, η_p_^2^ = 0.035, and sign (*F*(1, 297) = 13.837, *p* < 0.001, η_p_^2^ = 0.045, although significant Bundle Size × Sign, *F*(2, 594) = 4.765, *p* = 0.009, η_p_^2^ = 0.016, and Bundle Size × Order, *F*(2, 594) = 3.304, *p* = 0.037, η_p_^2^ = 0.011 interactions were also observed (see Figure 4). No other main effects or interactions were significant (in all cases, *Fs* < 2.609, *ps* > 0.106; see Supplementary Material for complete reporting of nonsignificant results). Following ANCOVA, Bonferroni-adjusted *post hoc* comparisons were conducted to further investigate the significant Bundle Size × Sign and Bundle Size × Order interactions.

#### Bundle Size × Sign

Analysis of within-subject comparisons revealed significantly higher indifference points in the bundle-size 3 and 9 conditions compared to the bundle-size 1 condition in both the gains and losses groups (in all cases, *ps* < 0.001). No significant differences were observed between bundle-sizes 3 and 9 in either the gains or losses groups (in both cases, *ps* > 0.174). Analysis of between-subject comparisons revealed significantly higher indifference points in the losses compared to the gains group at bundle-size 3 and 9 (in both cases, *ps* < 0.001), but not bundle-size 1 (control), *p* = 0.179.

#### Bundle Size × Order

Analysis of within-subject comparisons revealed significantly higher indifference points in the bundle-size 3 and 9 conditions compared to the bundle-size 1 condition in both the ascending and descending orders (in all cases, *ps* < 0.001). No significant differences were observed between bundle-sizes 3 and 9 in either the ascending or descending orders (in both cases, *ps* > 0.270). Analysis of between-subject comparisons revealed no significant differences in indifference points between ascending and descending orders at any bundle size (in all cases, *ps* > 0.174).

### Sensitivity Analysis: Data Quality Checks

A total of 29 participants (9.60% of the sample) failed one or more of the three quality control questions in the choice bundling assessment (*n* = 7, 4, 8, and 10 in the gains/ascending, gains/descending, losses/ascending, and losses/descending groups, respectively). In logistic regression, the odds of this response type did not differ significantly between the gains and losses groups, OR = 1.177 (0.404, 3.427), *p* = 0.765, ascending and descending order groups, OR = 0.548 (0.153, 1.954), *p* = 0.354, or their interaction, OR = 2.353 (0.469, 11.796), *p* = 0.298.

In a sensitivity analysis, the ANCOVA described above was repeated when excluding these 29 participants. Briefly, all conclusions for main effects, interactions, and adjusted *post hoc* comparisons were consistent with those from the primary analysis. Full results are provided in Supplementary Material Section 2.2.

### Sensitivity Analysis: Unrealistic Delays

Due to the use of participant-specific ED50 values as intervals between consecutive gains/losses in the adjusting-amount task, participants with high ED50 values encountered delays to one or more bundled consequences that exceeded their expected lifespan. For example, if a participant’s ED50 value in the six-trial, adjusting delay task were approximately 20 years, then any delayed gain or loss beyond the third in the bundle-size 9 condition would feature a delay exceeding 60 years (e.g., 20 years × 4 = 80). We thus explored the frequency with which participants encountered these unrealistic delays and their possible influence on outcomes.

Taking a conservative approach, we identified all participants exposed to a maximum delay in the choice bundling assessment (i.e., the final gain or loss at bundle-size 9, or ED50 × 9) that exceeded their expected remaining life years, given their current age. Expected remaining life years for the United States general population were collected from data reported by the National Center for Health Statistics and the Centers for Disease Control (Arias, 2021), and ranged from 56.9 years for 20-year-olds to 9.1 years for 80-year-olds. A total of *n* = 57 such participants (18.87% of the sample) were identified who met this criterion (*n* = 6, 5, 18, and 28 in the gains/ascending, gains/descending, losses/ascending, and losses/descending groups, respectively). In logistic regression, the odds of this response type was significantly greater in the losses compared to gain groups, OR = 3.684 (1.372, 9.894), *p* = 0.010, but did not differ significantly between order groups, OR = 0.822 (0.240, 2.816), *p* > 0.755, or the Sign × Order interaction, OR = 2.296 (0.555, 9.502), *p* = 0.251.

In a sensitivity analysis, we repeated the ANCOVA described above when excluding these 57 participants. Briefly, conclusions for main effects and interactions were consistent with those from the primary analysis, with the following exception: the effect of ethnicity was significant, *F*(1, 240) = 4.357, *p* = 0.038, η_p_^2^ = 0.018, with lower indifference points observed for Hispanic/Latino compared to other participants; however, ethnicity did not significantly interact with bundle size, *F*(2, 480) = 1.277, *p* = 0.280, η_p_^2^ = 0.005. Conclusions from adjusted post-hoc comparisons were also consistent with those from the primary analysis, with the following exception. Indifference points in the losses group were significantly higher compared to the gains group at bundle-size 1 (*p* = 0.035). Full results are provided in Supplementary Material, Section 2.3.

## Discussion

Data from the present study replicate prior findings in which choice bundling increases valuation of delayed gains (Kirby and Guastello, 2001; Ainslie and Monterosso, 2003; Hofmeyr et al., 2011; Stein et al., 2013; Stein and Madden, 2021), and extend these findings by showing that choice bundling produces even larger increases in valuation of delayed losses. This interaction between choice bundling and sign (gain vs. loss) was evident in the primary analysis including all data as well as in sensitivity analyses excluding participants with potentially poor quality data and unrealistic delays. Likewise, adjusted post-hoc comparisons generally revealed larger effects of choice bundling for losses compared to gains. The effects of bundle size also significantly interacted with bundle-size order (ascending vs. descending) in all analyses. Importantly, however, we observed no significant three-way interaction between bundle size, sign, and order, indicating that the differential effects of choice bundling for losses compared to gains did not depend on order.

### Choice Bundling and the Sign Effect

In choices for unbundled outcomes, losses are reliably discounted at a lower rate than gains (Thaler, 1981; Benzion et al., 1989; Murphy et al., 2001; Estle et al., 2006; Tanaka et al., 2014), meaning that individuals are typically more likely to minimize losses in intertemporal choice (by preferring the SS loss) than to maximize gains (by preferring the LL gain). Indeed, this “sign effect” for unbundled outcomes was replicated in the present study in the initial assessment of discounting (see Figure 3). Although the mechanisms underlying this gain–loss asymmetry are not well understood, some have argued (e.g., Loewenstein and Prelec, 1992) that it may emerge from the phenomenon of loss aversion, in which losses exert greater influence on choice than equivalently sized gains (Kahneman and Tversky, 1979; Tversky and Kahneman, 1991; Rasmussen and Newland, 2008). Loss aversion may, in turn, interact with the “magnitude effect,” in which larger amounts are discounted at a lower rate than smaller amounts (Thaler, 1981; Kirby and Maraković, 1995; Green et al., 1997; Mellis et al., 2017). Because losses are subjectively valued more highly than gains, the sign effect in intertemporal choice may be a special instance of the magnitude effect (e.g., Loewenstein and Prelec, 1992).

The sign effect may also be the result of feelings of dread and anticipatory anxiety experienced when waiting for losses (Berns et al., 2006; Hardisty and Weber, 2019; Molouki et al., 2019). As others have noted (e.g., Hardisty and Weber, 2020), waiting for gains is a multi-dimensional experience in which individuals may enjoy imagining the delayed, positive outcomes while also disliking having to wait for them. In contrast, waiting for losses or other aversive events is unidimensional, in which individuals dislike both the outcomes and having to wait for them, producing greater motivation to escape that aversive emotional state and “get it over with.”

The present study is the first to demonstrate that the sign effect is also evident in intertemporal choice for bundled outcomes. That is, significant interactions between bundle size and sign were observed in all analyses. Interestingly, in using the present study’s two-step adaptive procedures, this asymmetry in bundling effects was evident even when controlling for baseline differences in discounting of gains and losses; that is, in analyses in which indifference points in the bundle-size 1 control did not significantly differ between gains and losses (see Figure 4 and Supplementary Figure 1). Only in one sensitivity analysis (see Supplementary Figure 2) were significant differences between losses and gains observed in the bundle-size 1 control condition, although the Bundle Size × Sign interaction nonetheless remained significant in that analysis.

### Choice Bundling and the Preference Reversal Effect for Unbundled Outcomes

Choice bundling effects are related to another set of experimental findings, the “preference reversal” effect, in which adding a delay to both choice options can shift preference between unbundled SS and LL outcomes (for review, see Madden and Johnson, 2010). For example, one may prefer an immediate SS reward over a LL reward (e.g., $500 now vs. $1000 in 1 year), but switch their preference to the LL reward as the delays to *both* options increase (e.g., $500 in 1 year vs. $1,000 in 2 years).

Both the preference reversal effect for unbundled choices and choice bundling effects emerge from predictions of hyperbolic delay discounting. Specifically, in the preference reversal effect, adding an equal delay to both choice options of sufficient length (depending on individual delay discounting rate; Pope et al., 2019) allows the hyperbolic discounted value curve of the LL option to transect and exceed that of the now-discounted SS option, resulting in greater preference for LL gains (e.g., Rachlin and Green, 1972; Green et al., 1994; Pope et al., 2019) and SS losses (Holt et al., 2008). Notably, choice bundling manipulations also involve adding delays to each sequential pair of SS and LL outcomes (e.g., see Figure 2). For example, in the present study, the second SS and LL outcome in the bundling condition occurred at ED50 and 2*ED50, respectively; the third SS and LL outcomes occurred at 2*ED50 and 3*ED50, respectively; and so on (see Figure 2). Thus, the greater relative value of the LL option in each of these outcome pairs accumulates incrementally in the summed subjective value of the LL option to exceed that of the SS option. In this way, choice bundling effects leverage hyperbolic discounting to influence choice.

### Potential Clinical Utility of Choice Bundling

Prior research demonstrates that high rates of delay discounting predict initiation of cigarette smoking (Audrain-McGovern et al., 2009), differentiate smokers from non-smokers and former smokers (Odum et al., 2002; Stein et al., 2018a), are associated with greater addiction severity (Johnson et al., 2007; Sweitzer et al., 2008), and predict relapse following cessation (Yoon et al., 2007; Sheffer et al., 2014). These findings establish delay discounting as a potential therapeutic target (Riddle and Science of Behavior Change Working Group, 2015), in which interventions that reduce delay discounting may also facilitate smoking cessation (e.g., by increasing the relative value of long-term good health compared to immediate nicotine reinforcement). Preliminary experimental evidence further supports this view, as an intervention that guides individuals to engage in episodic prospection (i.e., to simulate future events) reduces delay discounting, cigarette smoking, and economic valuation of cigarettes (Stein et al., 2016, 2018b; Chiou and Wu, 2017).

Given these findings, development of additional methods to mitigate the possible role of high discounting rates in smoking behavior may yield efficacious treatments for cessation. Choice bundling has been shown in several studies to increase adaptive preference for LL gains (for review, see Ashe and Wilson, 2020) and, in the present study, SS losses. However, the majority of these studies employed precise control over the timing and magnitude of behavioral consequences in order to observe these effects. In contrast, in clinical contexts, manipulation of the natural consequences of cigarette smoking is not possible. For example, a treatment provider cannot control when or how frequently a patient may experience the health losses associated with continued smoking or health gains associated with cessation. For this reason, adaptation of laboratory-based choice bundling methods is necessary before attempts at clinical application. Two prior laboratory studies (Kirby and Guastello, 2001; Hofmeyr et al., 2011) have shown that a bundling-focused framing intervention, in which experimenters suggested to participants’ that their current choices were predictive of future choices (and are, therefore, bundled) increases preference for LL gains. Although this framing intervention has not been evaluated clinically to our knowledge, these studies suggest that interventions that prompt individuals to view their decisions as a temporally extended pattern of behavior producing cumulative outcomes may promote more adaptive intertemporal choice.

Importantly, most of the negative health consequences of cigarette smoking (e.g., COPD) are chronic conditions in which bundled symptoms (e.g., impaired breathing, circulation, and stamina) are experienced and escalate over time. As suggested previously (Stein and Madden, 2021), bundling-focused, motivational interventions could therefore be designed to guide individuals to repeatedly evaluate (e.g., with every urge to smoke) the cumulative value of long-term health against the momentary value of nicotine reinforcement. Toward this end, evidence from the present study that choice bundling produces larger effects for losses compared to gains may be critical. That is, gain-loss asymmetry in choice bundling suggests that attempts to develop bundling-focused clinical interventions for smoking cessation may take advantage of the sign effect by emphasizing the negative consequences of continued smoking as opposed to the positive consequences of smoking cessation. However, further research is required to determine whether the sign effect in choice bundling for monetary outcomes generalizes to other commodities, such as hypothetical health (e.g., Odum et al., 2002). Likewise, further research should examine whether the effects of choice bundling are observed during nicotine withdrawal, as prior research shows that 12 h of smoking abstinence increases delay discounting (Heckman et al., 2017) and this nicotine-deprived state may more closely approximate the clinical environment in which individuals are attempting to abstain from smoking.

### Potential Limitations

A few limitations of the present study deserve note. First, use of the adjusting-amount task to generate a single indifference point may have limited resolution to detect effects of choice bundling. The adjusting-amount task is most commonly used to assess indifference points across a range of delays (e.g., 1 month to 20 years; Du et al., 2002), yielding a full discounting curve, from which high-resolution estimates of discounting may be derived. In contrast, assessment of only a single indifference point yields a less granular estimate of discounting and may have resulted in a ceiling effect that diminished sensitivity to detect differences between bundle-sizes 3 and 9, as differences in intertemporal choice for gains between these conditions have been shown in prior studies using alternative methods (Stein et al., 2013; Stein and Madden, 2021).

Second, use of the two-step, adaptive procedure in which we assessed ED50 using the 6-trial, adjusting-delay task followed by assessment of indifferent points using the adjusting-amount task, limited the ability to examine concordance between observed effects and those predicted by individual participants’ *k* values in the additive hyperbolic discounting model (Equation 2), as done previously (Stein et al., 2013; Stein and Madden, 2021). Specifically, if both tasks produced perfectly concordant estimates of choice, then assessment at the bundle-size 1 (control) condition should have yielded indifference points at $450 (half of $900). In contrast, mean indifference points in this condition were all above $540, regardless of sign or order. This suggests that the six-trial task overestimates *k* (i.e., underestimates ED50) relative to the adjusting-amount task. This is consistent with prior evidence that the similar five-trial, adjusting-delay task also produces higher estimates of *k* than the adjusting-amount task, despite strong correlations between tasks (e.g., *r* = 0.67–0.86 Koffarnus and Bickel, 2014; Stein et al., 2017). In future studies, researchers should consider the use of only a single task to minimize measurement error.

Third, as a result of the method in which choice bundling was arranged, approximately 19% of the sample were exposed to one or more delays in the adjusting-amount task that likely exceeded their expected lifespan. This did not substantially impact our conclusions, as a sensitivity analysis excluding these participants revealed largely similar effects as the primary analysis. Nonetheless, in future studies, researchers may explore use of alternative delays and intervals between bundled consequences (e.g., half of ED50) in order to reduce or eliminate the probability of exposure to these unrealistic delays. Alternatively, as suggested previously (Stein et al., 2013; Stein and Madden, 2021), bundle size could be limited to no more than three gains or losses to reduce the cumulative delay period. This is unlikely to substantially limit effect sizes because observed effects of choice bundling and those predicted by Equation 2 are largest at smaller bundle sizes, with larger bundle sizes subject to diminishing marginal efficacy (for discussion, see Stein and Madden, 2021).

Fourth, although recruitment of opt-in, online panels in addiction science can provide useful and generalizable evidence in decision-making research (Strickland and Stoops, 2019; Mellis and Bickel, 2020), it often yields participant samples that are not representative of the broader population of interest. This was true in the present study, in which college-educated adults were over-represented and minorities were under-represented compared to prevalence in the United States population of smokers. As such, future research should examine the generality of the effects observed here in more diverse, nationally representative samples. Moreover, despite use of random allocation and minimal attrition, groups were not balanced on ethnicity (90.0 and 79.61% Non-Hispanic/Latino in the gains and losses groups, respectively). This imbalance contributed unwanted variability in choice estimates and may have reduced effect sizes. Nonetheless, effects of choice bundling were evident even when including this relatively minor difference in ethnicity as a covariate and, importantly, ethnicity did not significantly interact with bundle size, sign, or order to influence choice in the assessment of choice bundling.

## Conclusion

We conclude that choice bundling increases valuation of both delayed monetary gains and losses in cigarette smokers, although effects for losses are larger compared to gains. Future research should examine whether choice bundling effects generalize to non-monetary, health outcomes and whether choice bundling can lead to efficacious interventions for smoking cessation.

## Data Availability Statement

The datasets presented in this study can be found in online repositories. The names of the repository/repositories and accession number(s) can be found below: https://osf.io/63xjr/?view_only=3e8c00292e8e46ec87866bcc69443c01.

## Ethics Statement

The studies involving human participants were reviewed and approved by Virginia Tech Institutional Review Board. Written informed consent for participation was not required for this study in accordance with the national legislation and the institutional requirements.

## Author Contributions

JS and GM contributed to conception and design of the study. RF-L organized recruitment of the participant sample with Ipsos. RF-L and JS created the survey instruments. JS and JB performed the statistical analyses, with input from AT. JS and JB wrote the first draft of the manuscript. All authors contributed to manuscript revision, read, and approved the submitted version.

## Conflict of Interest

Although the following activities/relationships do not create a conflict of interest pertaining to this manuscript, in the interest of full disclosure, the authors would like to report the following: WB is a principal of HealthSim, LLC; BEAM Diagnostics, Inc.; and Red 5 Group, LLC. In addition, he serves on the scientific advisory board for Sober Grid, Inc.; and Ria Health; serves as a consultant for Boehringer Ingelheim International; and works on a project supported by Indivior, Inc. JS has received funding from the National Institutes of Health through a subcontract awarded to BEAM Diagnostics, Inc. The remaining authors declare that the research was conducted in the absence of any commercial or financial relationships that could be construed as a potential conflict of interest.

## Publisher’s Note

All claims expressed in this article are solely those of the authors and do not necessarily represent those of their affiliated organizations, or those of the publisher, the editors and the reviewers. Any product that may be evaluated in this article, or claim that may be made by its manufacturer, is not guaranteed or endorsed by the publisher.

## Footnotes

1
social.i-say.com

2
innovatemr.com/panels/consumer-panel
