# Supplementary material for: Choice Bundling Increases Valuation of Delayed Losses More Than Gains in Cigarette Smokers
Source: Front Behav Neurosci. 2022 Jan 13;15:796502. doi: 10.3389/fnbeh.2021.796502 (PMC8793342; doi:10.3389/fnbeh.2021.796502)
Supplement: Supplementary file 1 [file Data_Sheet_1.DOCX]

***Supplementary Material***

**1. Supplementary Procedures**

**1.1 Six-Trial, Adjusting-Delay Task (Step 1)**

Participants were presented with six hypothetical choices between receiving or losing (depending on group) either a larger amount ($900) after a delay or half of this amount ($450) immediately. The delay to the LL amount started at 1 day on the first trial and was adjusted following each trial, based on the preceding choice. Specifically, in the gains task, choices for the larger amount increased the delay, and choices for the smaller amount decreased the delay, on the next trial; in the losses task, this relationship was reversed. The adjusted value after the final trial was the delay expected to produce indifference between options and provided a measure of ED50 (in days), one of 64 possible values ranging from 4 seconds to 90 years in approximately logarithmic intervals. Supplementary Tables 1 and 2 provide the branching logic for delay adjustment across trials in the gains and losses tasks, respectively. Also depicted is scoring of ED50 and *k*.

Prior to the assigned task, participants read the following instructions. Bracketed text varied depending on group assignment (gains vs. losses).

You will now be presented with a series of choices between [receiving/losing] different amounts of money at different points in time (e.g., [as a prize or gift/unavoidable fine or tax]). Please assume that the options you choose are guaranteed to happen (for sure) and the amounts will be automatically [deposited to/withdrawn from] your checking, savings, or other financial accounts at the time specified in each question.

These questions are hypothetical, but please choose your answer as if you will [receive/lose] the money in the time frame(s) selected. Please pay close attention to the amount and time frame(s) of each option, and choose accordingly. There are no right or wrong answers in this task. Please take your time.

**1.2 Adjusting-Amount Task (Step 2)**

Prior to the task, participants read the following instructions. Bracketed text varied depending on both group assignment (gains vs. losses) and individual ED50 values.

You will now again be presented with a series of choices between [receiving/losing] different amounts of money at different points in time (e.g., [as a prize or gift/as an unavoidable fine or tax]). Please assume that the options you choose are guaranteed to happen (for sure) and the amounts will be automatically [deposited to/withdrawn from] your checking, savings, or other financial accounts at the time specified in each question.

In some questions, the amounts will be [delivered/lost] all at once in lump sums. For example, you may see a question like this:

*Which would you rather [receive/lose]?*

*A. $450 now*

*or*

*B. $900 in [ED50]*

In other questions, these amounts will be [*delivered/lost*] in installments over time. For example, you may see a question like this:

*Which would you rather [receive/lose]?*

*A. $150 now... another $150 in [ED50]... and another $150 in [ED50*2]*

*or*

*B. $300 in [ED50]... another $300 in [ED50*2]... and another $300 in [ED50*3]*

 These questions are hypothetical, but please choose your answer as if you will [receive/lose] the money in the time frame(s) selected. Please pay close attention to the amount and time frame(s) of each option, and choose accordingly. There are no right or wrong answers in this task. Please take your time.

**2. Supplementary Results**

**2.1 Primary Analysis**

Significant main effects and interaction are reported in main text. Nonsignificant effects include the main effect of order, *F*(1, 297)=0.001, *p*=.993, 𝜂_p_^2^=.000, and ethnicity, *F*(1, 297)=2.608, *p*=.107, 𝜂_p_^2^=.009; and Bundle Size x Ethnicity interaction, *F*(2, 594)=1.096, *p*=.335, 𝜂_p_^2^=.004, and Bundle Size x Sign x Order interaction, *F*(2, 594)=0.376, *p*=.683, 𝜂_p_^2^=.001.

**2.2 Sensitivity Analysis: Data Quality Checks**

In a sensitivity analysis excluding the 29 participants who failed one or more quality control questions, ANCOVA revealed significant main effects of bundle size, *F*(2, 536)=9.766, *p*<.001, 𝜂_p_^2^=.035, and sign (*F*(1, 268)=18.955, *p*<.001, 𝜂_p_^2^=.066, as well as a significant Bundle Size x Sign interaction, *F*(2, 536)=7.157, *p*<.001, 𝜂_p_^2^=.026, and Bundle Size x Order interaction, *F*(2, 536)=3.304, *p*=.019, 𝜂_p_^2^=.015 (see Figure S1). No other main effects or interactions were significant, including the main effect of order, *F*(1, 268)=0.826, *p*=.364, 𝜂_p_^2^=.003, and ethnicity, *F*(1, 268)=.217, *p*=.642, 𝜂_p_^2^=.001; and Bundle Size x Ethnicity interaction, *F*(2, 536)=0.574, *p*=.564, 𝜂_p_^2^=.002, and Bundle Size x Sign x Order interaction, *F*(2, 536)=0.191, *p*=.819, 𝜂_p_^2^=.001.

Following ANCOVA, pairwise comparisons were conducted to further investigate the significant Bundle Size x Sign and Bundle Size x Order interactions.

*Bundle Size x Sign.* Analysis of within-subject comparisons revealed significantly higher indifference points in the bundle-size 3 and 9 conditions compared to the bundle-size 1 condition in both the gains and losses groups (in all cases, *p*<.001). No significant differences were observed between bundle-sizes 3 and 9 in either the gains or losses groups (in both cases, *p*>.198). Analysis of between-subject comparisons revealed significantly higher indifference points in the losses compared to the gains group at bundle-size 3 and 9 (in both cases, *p*<.001), but not bundle-size 1 (control), *p*=.204.

*Bundle Size x Order.* Analysis of within-subject comparisons revealed significantly higher indifference points in the bundle-size 3 and 9 conditions compared to the bundle-size 1 condition in both the ascending and descending orders (in all cases, *p*<.001). No significant differences were observed between bundle-sizes 3 and 9 in either the ascending or descending orders (in both cases, *p*>.421). Analysis of between-subject comparisons revealed no significant differences in indifference points between ascending and descending orders at any bundle size (in all cases, *p*>.084).

**2.3 Sensitivity Analysis: Unrealistic Delays**

In a sensitivity analysis excluding the 57 participants who were exposed to a maximum delay that exceeded their expected remaining life years, ANCOVA revealed significant main effects of bundle size, *F*(2, 480)=8.772, *p*<.001, 𝜂_p_^2^=.035, and sign (*F*(1, 240)=22.003, *p*<.001, 𝜂_p_^2^=.084, as well as a significant Bundle Size x Sign interaction, *F*(2, 480)=3.646, *p*=.027, 𝜂_p_^2^=.015, and Bundle Size x Order interaction, *F*(2, 480)=4.527, *p*=.011, 𝜂_p_^2^=.019 (see Figure S2). The effect of ethnicity was also significant, *F*(1, 240)=4.357, *p*=.038, 𝜂_p_^2^=.018No other main effects or interactions were significant, including the main effect of order, *F*(1, 240)=0.498, *p*=.481, 𝜂_p_^2^=.002, and Bundle Size x Ethnicity interaction, *F*(2, 480)=1.277, *p*=.280, 𝜂_p_^2^=.005, and Bundle Size x Sign x Order interaction, *F*(2, 480)=0.310 *p*=.733, 𝜂_p_^2^=.001.

Following ANCOVA, pairwise comparisons were conducted to further investigate the significant Bundle Size x Sign and Bundle Size x Order interactions.

*Bundle Size x Sign.* Analysis of within-subject comparisons revealed significantly higher indifference points in the bundle-size 3 and 9 conditions compared to the bundle-size 1 condition in both the gains and losses groups (in all cases, *p*<.001). No significant difference was observed between bundle-size 3 and 9 conditions in the gains or losses group (in both cases, *p*>.068). Analysis of between-subject comparisons revealed significantly higher indifference points in the losses compared to the gains group at bundle-size 1, 3, and 9 (in all cases, *p*<.05).

*Bundle Size x Order.* Analysis of within-subject comparisons revealed significantly higher indifference points in the bundle-size 3 and 9 conditions compared to the bundle-size 1 condition in both the ascending and descending orders (in all cases, *p*<.001). No significant differences were observed between bundle-sizes 3 and 9 in either the ascending or descending orders (in both cases, *p*>.352). Analysis of between-subject comparisons revealed significantly higher indifference points in the descending compared to ascending order at bundle-size 1 (*p=*.025); no significant differences in indifference points between ascending and descending orders were observed at any other bundle size (in all cases, *p*>.698).

**3. Supplementary Tables**

Supplementary Tables 1 and 2 provide the branching logic for delay adjustment in the 6-trial, adjusting-delay task for gains and losses, respectively. ED50 is scored as the adjusted delay after the final trial. Note that because the smaller amount in this task is half of the larger amount, *k* may also be calculated as the inverse of ED50 (i.e., 1/ED50; Koffarnus and Bickel 2014; Yoon and Higgins 2008).

*Supplementary Table 1.* Branching logic and scoring of ED50/*k* in the gains version of the 6-trial, adjusting-delay task.

| **Gains task** | | | | | | | | |
| --- | --- | --- | --- | --- | --- | --- | --- | --- |
| **Administration** | | | | | **Scoring** | | | |
| **Trial #** | **Choice options (A=Amount)** | **If 0.5A chosen, branch to index #…** | **If A chosen, branch to index #…** | **Index #** | **ED50 (days) if**  **0.5A chosen** | **ED50 (days) if**  **A chosen** | ***k* if**  **0.5A chosen** | ***k* if**  **A chosen** |
| 1 | Receive [0.5A] now vs. [A] in 1 day | 16 | 48 | 32 | . | . | . | . |
| 2 | Receive [0.5A] now vs. [A] in 9 minutes | 8 | 24 | 16 | . | . | . | . |
| 2 | Receive [0.5A] now vs. [A] in 8 months | 40 | 56 | 48 | . | . | . | . |
| 3 | Receive [0.5A] now vs. [A] in 30 seconds | 4 | 12 | 8 | . | . | . | . |
| 3 | Receive [0.5A] now vs. [A] in 2 hours | 20 | 28 | 24 | . | . | . | . |
| 3 | Receive [0.5A] now vs. [A] in 2 weeks | 36 | 44 | 40 | . | . | . | . |
| 3 | Receive [0.5A] now vs. [A] in 9 years | 52 | 60 | 56 | . | . | . | . |
| 4 | Receive [0.5A] now vs. [A] in 10 seconds | 2 | 6 | 4 | . | . | . | . |
| 4 | Receive [0.5A] now vs. [A] in 2 minutes | 10 | 14 | 12 | . | . | . | . |
| 4 | Receive [0.5A] now vs. [A] in 30 minutes | 18 | 22 | 20 | . | . | . | . |
| 4 | Receive [0.5A] now vs. [A] in 7 hours | 26 | 30 | 28 | . | . | . | . |
| 4 | Receive [0.5A] now vs. [A] in 4 days | 34 | 38 | 36 | . | . | . | . |
| 4 | Receive [0.5A] now vs. [A] in 2 months | 42 | 46 | 44 | . | . | . | . |
| 4 | Receive [0.5A] now vs. [A] in 2 years | 50 | 54 | 52 | . | . | . | . |
| 4 | Receive [0.5A] now vs. [A] in 33 years | 58 | 62 | 60 | . | . | . | . |
| 5 | Receive [0.5A] now vs. [A] in 5 seconds | 1 | 3 | 2 | . | . | . | . |
| 5 | Receive [0.5A] now vs. [A] in 20 seconds | 5 | 7 | 6 | . | . | . | . |
| 5 | Receive [0.5A] now vs. [A] in 1 minute | 9 | 11 | 10 | . | . | . | . |
| 5 | Receive [0.5A] now vs. [A] in 4 minutes | 13 | 15 | 14 | . | . | . | . |
| 5 | Receive [0.5A] now vs. [A] in 17 minutes | 17 | 19 | 18 | . | . | . | . |
| 5 | Receive [0.5A] now vs. [A] in 1 hour | 21 | 23 | 22 | . | . | . | . |
| 5 | Receive [0.5A] now vs. [A] in 4 hours | 25 | 27 | 26 | . | . | . | . |
| 5 | Receive [0.5A] now vs. [A] in 15 hours | 29 | 31 | 30 | . | . | . | . |
| 5 | Receive [0.5A] now vs. [A] in 2 days | 33 | 35 | 34 | . | . | . | . |
| 5 | Receive [0.5A] now vs. [A] in 1 week | 37 | 39 | 38 | . | . | . | . |
| 5 | Receive [0.5A] now vs. [A] in 1 month | 41 | 43 | 42 | . | . | . | . |
| 5 | Receive [0.5A] now vs. [A] in 4 months | 45 | 47 | 46 | . | . | . | . |
| 5 | Receive [0.5A] now vs. [A] in 1 year | 49 | 51 | 50 | . | . | . | . |
| 5 | Receive [0.5A] now vs. [A] in 5 years | 53 | 55 | 54 | . | . | . | . |
| 5 | Receive [0.5A] now vs. [A] in 17 years | 57 | 59 | 58 | . | . | . | . |
| 5 | Receive [0.5A] now vs. [A] in 65 years | 61 | 63 | 62 | . | . | . | . |
| 6 | Receive [0.5A] now vs. [A] in 4 seconds | . | . | 1 | 4.62963E-05 | 0.00005787 | 21600 | 17280.11059 |
| 6 | Receive [0.5A] now vs. [A] in 7 seconds | . | . | 3 | 0.000068473 | 0.000096836 | 14604.29658 | 10326.73799 |
| 6 | Receive [0.5A] now vs. [A] in 15 seconds | . | . | 5 | 0.000141753 | 0.000200469 | 7054.524419 | 4988.302431 |
| 6 | Receive [0.5A] now vs. [A] in 25 seconds | . | . | 7 | 0.000258804 | 0.000316969 | 3863.927915 | 3154.882654 |
| 6 | Receive [0.5A] now vs. [A] in 45 seconds | . | . | 9 | 0.000425259 | 0.000601407 | 2351.50814 | 1662.767477 |
| 6 | Receive [0.5A] now vs. [A] in 1½ minutes | . | . | 11 | 0.000850517 | 0.001202813 | 1175.755452 | 831.3844297 |
| 6 | Receive [0.5A] now vs. [A] in 3 minutes | . | . | 13 | 0.001701035 | 0.002405626 | 587.8773805 | 415.6922148 |
| 6 | Receive [0.5A] now vs. [A] in 6 minutes | . | . | 15 | 0.003402069 | 0.005103104 | 293.9387767 | 195.9591652 |
| 6 | Receive [0.5A] now vs. [A] in 12 minutes | . | . | 17 | 0.007216878 | 0.009918651 | 138.5640716 | 100.8201619 |
| 6 | Receive [0.5A] now vs. [A] in 23 minutes | . | . | 19 | 0.01373175 | 0.018241563 | 72.82392994 | 54.81986385 |
| 6 | Receive [0.5A] now vs. [A] in 45 minutes | . | . | 21 | 0.025515518 | 0.036084392 | 39.19183612 | 27.71281279 |
| 6 | Receive [0.5A] now vs. [A] in 1½ hours | . | . | 23 | 0.051031036 | 0.072168784 | 19.59591806 | 13.85640639 |
| 6 | Receive [0.5A] now vs. [A] in 3 hours | . | . | 25 | 0.102062073 | 0.144337567 | 9.797958934 | 6.928203245 |
| 6 | Receive [0.5A] now vs. [A] in 5 hours | . | . | 27 | 0.186338998 | 0.246503324 | 5.36656315 | 4.056740428 |
| 6 | Receive [0.5A] now vs. [A] in 10 hours | . | . | 29 | 0.348608344 | 0.510310363 | 2.868548666 | 1.959591795 |
| 6 | Receive [0.5A] now vs. [A] in 20 hours | . | . | 31 | 0.721687836 | 0.912870929 | 1.385640647 | 1.095445115 |
| 6 | Receive [0.5A] now vs. [A] in 1½ days | . | . | 33 | 1.224744871 | 1.732050808 | 0.816496581 | 0.577350269 |
| 6 | Receive [0.5A] now vs. [A] in 3 days | . | . | 35 | 2.449489743 | 3.464101615 | 0.40824829 | 0.288675135 |
| 6 | Receive [0.5A] now vs. [A] in 6 days | . | . | 37 | 4.898979486 | 6.480740698 | 0.204124145 | 0.15430335 |
| 6 | Receive [0.5A] now vs. [A] in 1½ weeks | . | . | 39 | 8.5732141 | 12.12435565 | 0.116642369 | 0.08247861 |
| 6 | Receive [0.5A] now vs. [A] in 3 weeks | . | . | 41 | 17.1464282 | 25.283196 | 0.058321184 | 0.039551962 |
| 6 | Receive [0.5A] now vs. [A] in 1½ months | . | . | 43 | 37.28123389 | 52.72362658 | 0.026823147 | 0.018966829 |
| 6 | Receive [0.5A] now vs. [A] in 3 months | . | . | 45 | 74.56246777 | 105.4472532 | 0.013411573 | 0.009483414 |
| 6 | Receive [0.5A] now vs. [A] in 6 months | . | . | 47 | 149.1249355 | 210.8945063 | 0.006705787 | 0.004741707 |
| 6 | Receive [0.5A] now vs. [A] in 10 months | . | . | 49 | 272.2636369 | 333.4397997 | 0.003672911 | 0.002999042 |
| 6 | Receive [0.5A] now vs. [A] in 1½ years | . | . | 51 | 447.3380643 | 632.6315575 | 0.002235446 | 0.001580699 |
| 6 | Receive [0.5A] now vs. [A] in 3 years | . | . | 53 | 894.6761286 | 1414.607167 | 0.001117723 | 0.00070691 |
| 6 | Receive [0.5A] now vs. [A] in 6 years | . | . | 55 | 2000.556641 | 2684.028386 | 0.000499861 | 0.000372574 |
| 6 | Receive [0.5A] now vs. [A] in 12 years | . | . | 57 | 3795.789345 | 5216.813467 | 0.00026345 | 0.000191688 |
| 6 | Receive [0.5A] now vs. [A] in 25 years | . | . | 59 | 7529.821649 | 10491.00753 | 0.000132805 | 9.53197E-05 |
| 6 | Receive [0.5A] now vs. [A] in 46 years | . | . | 61 | 14230.69499 | 19817.96821 | 7.02706E-05 | 5.04593E-05 |
| 6 | Receive [0.5A] now vs. [A] in 90 years | . | . | 63 | 23741.25 | 32872.5 | 4.21208E-05 | 3.04206E-05 |

A represents the amount of the larger, delayed consequence

*Supplementary Table 2.* Branching logic and scoring of ED50/*k* in the losses version of the 6-trial, adjusting-delay task.

| **Losses Task** | | | | | | | | |
| --- | --- | --- | --- | --- | --- | --- | --- | --- |
| **Administration** | | | | | **Scoring** | | | |
| **Trial #** | **Choice options (A=Amount)** | **If 0.5A chosen, branch to index #…** | **If A chosen, branch to index #…** | **Index #** | **ED50 (days) if**  **0.5A chosen** | **ED50 (days) if**  **A chosen** | ***k* if**  **0.5A chosen** | ***k* if**  **A chosen** |
| 1 | Lose [0.5A] now vs. [A] in 1 day | 48 | 16 | 32 | . | . | . | . |
| 2 | Lose [0.5A] now vs. [A] in 9 minutes | 24 | 8 | 16 | . | . | . | . |
| 2 | Lose [0.5A] now vs. [A] in 8 months | 56 | 40 | 48 | . | . | . | . |
| 3 | Lose [0.5A] now vs. [A] in 30 seconds | 12 | 4 | 8 | . | . | . | . |
| 3 | Lose [0.5A] now vs. [A] in 2 hours | 28 | 20 | 24 | . | . | . | . |
| 3 | Lose [0.5A] now vs. [A] in 2 weeks | 44 | 36 | 40 | . | . | . | . |
| 3 | Lose [0.5A] now vs. [A] in 9 years | 60 | 52 | 56 | . | . | . | . |
| 4 | Lose [0.5A] now vs. [A] in 10 seconds | 6 | 2 | 4 | . | . | . | . |
| 4 | Lose [0.5A] now vs. [A] in 2 minutes | 14 | 10 | 12 | . | . | . | . |
| 4 | Lose [0.5A] now vs. [A] in 30 minutes | 22 | 18 | 20 | . | . | . | . |
| 4 | Lose [0.5A] now vs. [A] in 7 hours | 30 | 26 | 28 | . | . | . | . |
| 4 | Lose [0.5A] now vs. [A] in 4 days | 38 | 34 | 36 | . | . | . | . |
| 4 | Lose [0.5A] now vs. [A] in 2 months | 46 | 42 | 44 | . | . | . | . |
| 4 | Lose [0.5A] now vs. [A] in 2 years | 54 | 50 | 52 | . | . | . | . |
| 4 | Lose [0.5A] now vs. [A] in 33 years | 62 | 58 | 60 | . | . | . | . |
| 5 | Lose [0.5A] now vs. [A] in 5 seconds | 3 | 1 | 2 | . | . | . | . |
| 5 | Lose [0.5A] now vs. [A] in 20 seconds | 7 | 5 | 6 | . | . | . | . |
| 5 | Lose [0.5A] now vs. [A] in 1 minute | 11 | 9 | 10 | . | . | . | . |
| 5 | Lose [0.5A] now vs. [A] in 4 minutes | 15 | 13 | 14 | . | . | . | . |
| 5 | Lose [0.5A] now vs. [A] in 17 minutes | 19 | 17 | 18 | . | . | . | . |
| 5 | Lose [0.5A] now vs. [A] in 1 hour | 23 | 21 | 22 | . | . | . | . |
| 5 | Lose [0.5A] now vs. [A] in 4 hours | 27 | 25 | 26 | . | . | . | . |
| 5 | Lose [0.5A] now vs. [A] in 15 hours | 31 | 29 | 30 | . | . | . | . |
| 5 | Lose [0.5A] now vs. [A] in 2 days | 35 | 33 | 34 | . | . | . | . |
| 5 | Lose [0.5A] now vs. [A] in 1 week | 39 | 37 | 38 | . | . | . | . |
| 5 | Lose [0.5A] now vs. [A] in 1 month | 43 | 41 | 42 | . | . | . | . |
| 5 | Lose [0.5A] now vs. [A] in 4 months | 47 | 45 | 46 | . | . | . | . |
| 5 | Lose [0.5A] now vs. [A] in 1 year | 51 | 49 | 50 | . | . | . | . |
| 5 | Lose [0.5A] now vs. [A] in 5 years | 55 | 53 | 54 | . | . | . | . |
| 5 | Lose [0.5A] now vs. [A] in 17 years | 59 | 57 | 58 | . | . | . | . |
| 5 | Lose [0.5A] now vs. [A] in 65 years | 63 | 61 | 62 | . | . | . | . |
| 6 | Lose [0.5A] now vs. [A] in 4 seconds | . | . | 1 | 0.00005787 | 4.62963E-05 | 17280.11059 | 21600 |
| 6 | Lose [0.5A] now vs. [A] in 7 seconds | . | . | 3 | 0.000096836 | 0.000068473 | 10326.73799 | 14604.29658 |
| 6 | Lose [0.5A] now vs. [A] in 15 seconds | . | . | 5 | 0.000200469 | 0.000141753 | 4988.302431 | 7054.524419 |
| 6 | Lose [0.5A] now vs. [A] in 25 seconds | . | . | 7 | 0.000316969 | 0.000258804 | 3154.882654 | 3863.927915 |
| 6 | Lose [0.5A] now vs. [A] in 45 seconds | . | . | 9 | 0.000601407 | 0.000425259 | 1662.767477 | 2351.50814 |
| 6 | Lose [0.5A] now vs. [A] in 1½ minutes | . | . | 11 | 0.001202813 | 0.000850517 | 831.3844297 | 1175.755452 |
| 6 | Lose [0.5A] now vs. [A] in 3 minutes | . | . | 13 | 0.002405626 | 0.001701035 | 415.6922148 | 587.8773805 |
| 6 | Lose [0.5A] now vs. [A] in 6 minutes | . | . | 15 | 0.005103104 | 0.003402069 | 195.9591652 | 293.9387767 |
| 6 | Lose [0.5A] now vs. [A] in 12 minutes | . | . | 17 | 0.009918651 | 0.007216878 | 100.8201619 | 138.5640716 |
| 6 | Lose [0.5A] now vs. [A] in 23 minutes | . | . | 19 | 0.018241563 | 0.01373175 | 54.81986385 | 72.82392994 |
| 6 | Lose [0.5A] now vs. [A] in 45 minutes | . | . | 21 | 0.036084392 | 0.025515518 | 27.71281279 | 39.19183612 |
| 6 | Lose [0.5A] now vs. [A] in 1½ hours | . | . | 23 | 0.072168784 | 0.051031036 | 13.85640639 | 19.59591806 |
| 6 | Lose [0.5A] now vs. [A] in 3 hours | . | . | 25 | 0.144337567 | 0.102062073 | 6.928203245 | 9.797958934 |
| 6 | Lose [0.5A] now vs. [A] in 5 hours | . | . | 27 | 0.246503324 | 0.186338998 | 4.056740428 | 5.36656315 |
| 6 | Lose [0.5A] now vs. [A] in 10 hours | . | . | 29 | 0.510310363 | 0.348608344 | 1.959591795 | 2.868548666 |
| 6 | Lose [0.5A] now vs. [A] in 20 hours | . | . | 31 | 0.912870929 | 0.721687836 | 1.095445115 | 1.385640647 |
| 6 | Lose [0.5A] now vs. [A] in 1½ days | . | . | 33 | 1.732050808 | 1.224744871 | 0.577350269 | 0.816496581 |
| 6 | Lose [0.5A] now vs. [A] in 3 days | . | . | 35 | 3.464101615 | 2.449489743 | 0.288675135 | 0.40824829 |
| 6 | Lose [0.5A] now vs. [A] in 6 days | . | . | 37 | 6.480740698 | 4.898979486 | 0.15430335 | 0.204124145 |
| 6 | Lose [0.5A] now vs. [A] in 1½ weeks | . | . | 39 | 12.12435565 | 8.5732141 | 0.08247861 | 0.116642369 |
| 6 | Lose [0.5A] now vs. [A] in 3 weeks | . | . | 41 | 25.283196 | 17.1464282 | 0.039551962 | 0.058321184 |
| 6 | Lose [0.5A] now vs. [A] in 1½ months | . | . | 43 | 52.72362658 | 37.28123389 | 0.018966829 | 0.026823147 |
| 6 | Lose [0.5A] now vs. [A] in 3 months | . | . | 45 | 105.4472532 | 74.56246777 | 0.009483414 | 0.013411573 |
| 6 | Lose [0.5A] now vs. [A] in 6 months | . | . | 47 | 210.8945063 | 149.1249355 | 0.004741707 | 0.006705787 |
| 6 | Lose [0.5A] now vs. [A] in 10 months | . | . | 49 | 333.4397997 | 272.2636369 | 0.002999042 | 0.003672911 |
| 6 | Lose [0.5A] now vs. [A] in 1½ years | . | . | 51 | 632.6315575 | 447.3380643 | 0.001580699 | 0.002235446 |
| 6 | Lose [0.5A] now vs. [A] in 3 years | . | . | 53 | 1414.607167 | 894.6761286 | 0.00070691 | 0.001117723 |
| 6 | Lose [0.5A] now vs. [A] in 6 years | . | . | 55 | 2684.028386 | 2000.556641 | 0.000372574 | 0.000499861 |
| 6 | Lose [0.5A] now vs. [A] in 12 years | . | . | 57 | 5216.813467 | 3795.789345 | 0.000191688 | 0.00026345 |
| 6 | Lose [0.5A] now vs. [A] in 25 years | . | . | 59 | 10491.00753 | 7529.821649 | 9.53197E-05 | 0.000132805 |
| 6 | Lose [0.5A] now vs. [A] in 46 years | . | . | 61 | 19817.96821 | 14230.69499 | 5.04593E-05 | 7.02706E-05 |
| 6 | Lose [0.5A] now vs. [A] in 90 years | . | . | 63 | 32872.5 | 23741.25 | 3.04206E-05 | 4.21208E-05 |

A represents the amount of the larger, delayed consequence


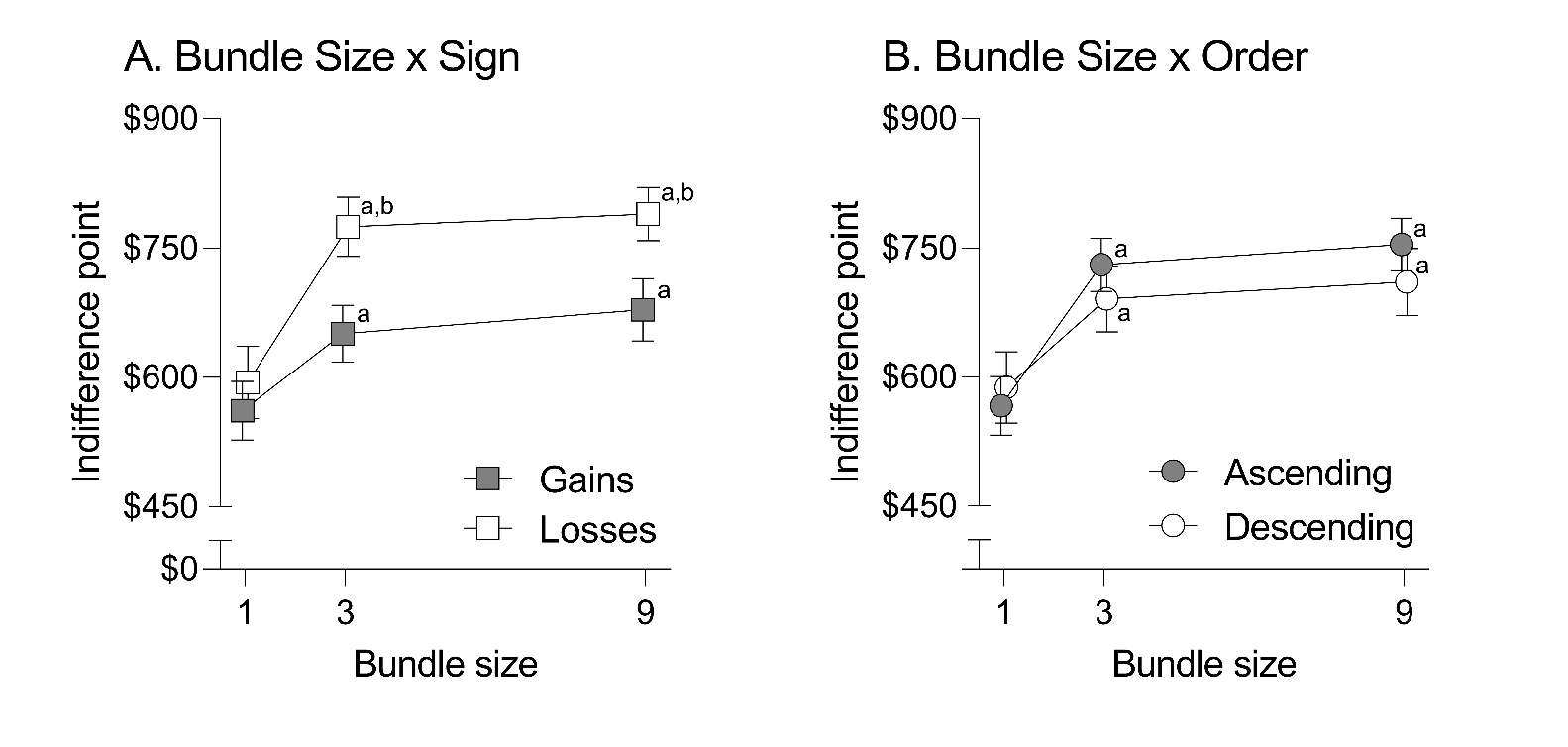


**Figure S1.** A sensitivity analysis in which 29 participants who failed one or more quality control questions were excluded. Depicted are significant effects of the Bundle Size x Sign (Panel A) and Bundle Size x Order (Panel B) interactions on indifference points in the adjusting-amount task. Error bars reflect 95% confidence intervals. Higher indifference points reflect greater valuation of the LL option. ^a^Significantly different from bundle-size 1 within the same sign or order group, *p*<.001. ^b^Significantly different from the opposing sign or order group at the same bundle size, *p*<.05.


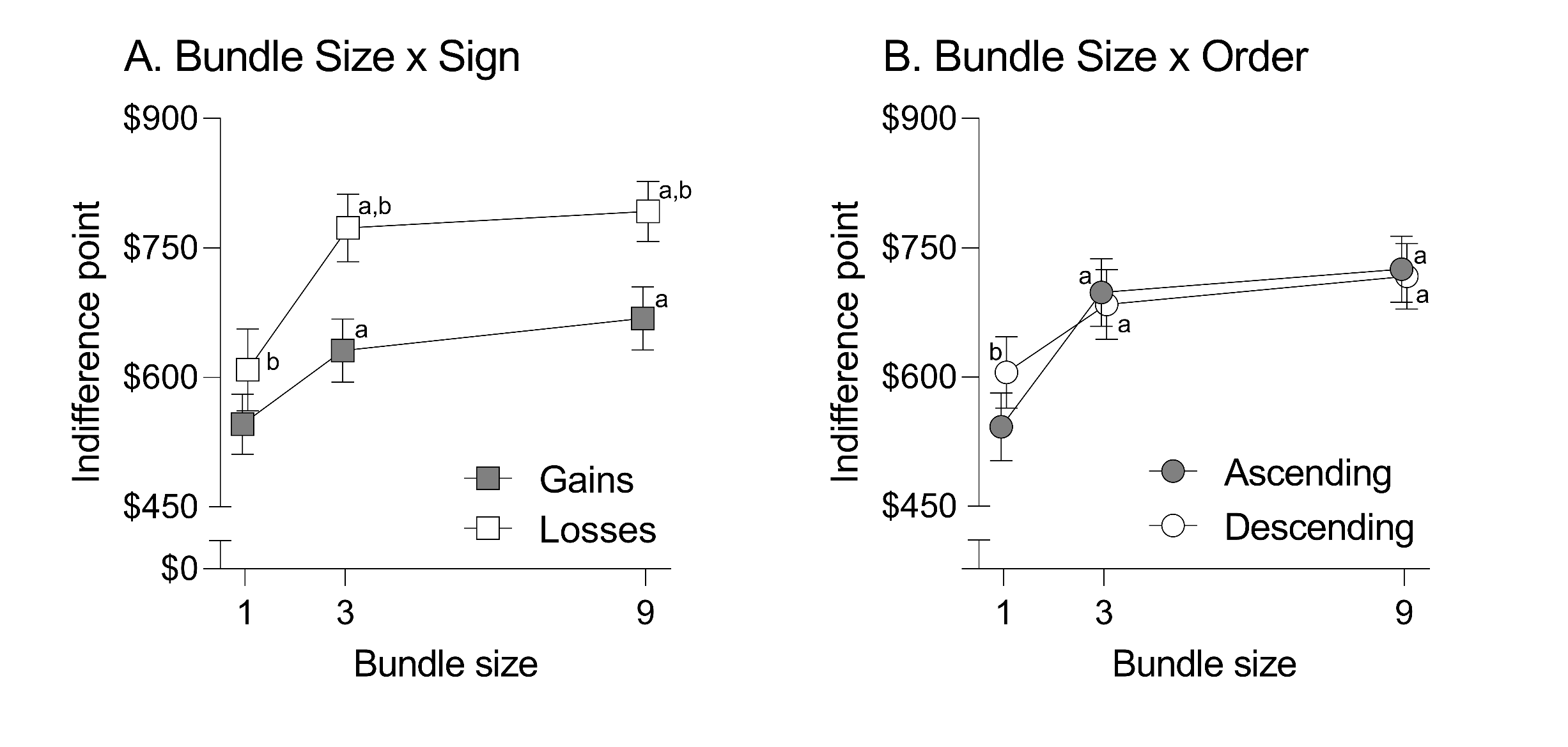


**Figure S2.** A sensitivity analysis in which 57 participants who were exposed to unrealistic delays in the choice bundling assessment were excluded. Depicted are significant effects of the Bundle Size x Sign (Panel A) and Bundle Size x Order (Panel B) interactions on indifference points in the adjusting-amount task. Error bars reflect 95% confidence intervals. Higher indifference points reflect greater valuation of the LL option. ^a^Significantly different from bundle-size 1 within the same sign or order group, *p*<.001. ^b^Significantly different from the opposing sign or order group at the same bundle size, *p*<.05.
